# Data-enabled Bayesian inference for strategic maintenance decisions in industrial operations

**DOI:** 10.1016/j.dib.2024.111058

**Published:** 2024-10-22

**Authors:** Raúl Torres-Sainz, Leandro L. Lorente-Leyva, Yorley Arbella-Feliciano, Carlos Alberto Trinchet-Varela, Lidia María Pérez-Vallejo, Roberto Pérez-Rodríguez

**Affiliations:** a CAD/CAM Study Center, University of Holguín, Holguín, Cuba; b SDAS Research Group (sdas-group.com/), Ben Guerir, 43150, Morocco; c Universidad UTE, Quito 170147, Ecuador; d Industrial Engineering Department, University of Holguín, Holguín, Cuba

**Keywords:** Maintenance management, Maintenance strategy selection, Intelligent predictive maintenance, Monte Carlo simulation

## Abstract

Efficient management of industrial assets and equipment depends heavily on the selection of appropriate maintenance strategies. This research presents a dataset generated through Monte Carlo simulations to evaluate 12 key criteria relevant to maintenance strategy selection. The dataset covers a wide range of potential maintenance scenarios, providing comprehensive data for researchers to explore various strategies in industrial settings. The data were normalized and structured in a way that facilitates their use for further modeling or analysis. The dataset offers an opportunity for researchers to reproduce the data collection process, enabling comparisons with their own studies. By providing this dataset, we aim to support the development of new models for maintenance strategy selection and encourage further exploration of data-driven approaches in industrial maintenance. Additionally, the dataset can serve educational purposes, assisting in the teaching of decision-making in the context of maintenance operations.

Specifications Table

**Table:** 

Subject	Engineering; Industrial Engineering; Mechanical Engineering
Specific subject area	Selecting maintenance strategies based on evaluating criteria and rules
Data format	Simulated and Raw
Type of data	Table
Data collection	The data article employed the Monte Carlo simulation method, utilizing the Excel function 'RANDOM.INBETWEEN' to assign random values to the 12 evaluated criteria and simulate various scenarios. A total of 1404 random scenarios were generated.
Data source location	University of Holguín, CAD/CAM Study Center, Holguín, Cuba
Data accessibility	Repository name: Mendeley Data [1] Data identification number: 10.17632/fgk42n93ks.2 Direct URL to data: https://data.mendeley.com/datasets/fgk42n93ks/2

## 1 Value of the Data

The data provided is undoubtedly highly valuable to the scientific community for several key reasons:

• 1. Detailed assessment: The data provides an in-depth assessment of 12 criteria related to maintenance strategies, enabling a thorough understanding of the many factors influencing decision making. The detailed analysis provided by these criteria facilitates the identification of interdependencies and priorities in maintenance planning, which is critical for effective asset and resource management.
• 2. Real-world relevance and benchmarking: The representation of real-world scenarios in the data makes it a valuable tool for researchers interested in practical and effective maintenance strategies. The inclusion of performance metrics such as corrective maintenance (CM), preventive maintenance (PM), preventive maintenance by condition status (PCSM), predictive maintenance (PdM) and intelligent predictive maintenance (IPdM) allows not only to understand the effectiveness of different maintenance approaches, but also to make direct comparisons between them, which is critical for continuous improvement of maintenance practices in real-world environments.

How other researchers can reuse this data:

• 1. Replication and Comparison: Other researchers can use these data as a benchmark to replicate the methods used in this data article and compare the results with their own research, which would encourage cross-validation of approaches and models in the field of industrial maintenance. This replication and comparison would contribute to strengthen the reliability of the methods used and enrich the discussion around the most effective maintenance strategies.
• 2. Development of New Models: Based on the data and methods presented, other researchers can develop and test new models for the selection of maintenance strategies, thus enriching the body of knowledge in this key area of industrial management. The possibility of generating new models would bring innovation and deepening in the field of maintenance, opening new perspectives for its optimization in diverse industrial environments.
• 3. Educational Purposes: The dataset can be used for educational purposes to teach decision making in maintenance operations based on empirical data. This educational use would not only facilitate the understanding of complex concepts, but also prepare future professionals for the practical application of advanced methods in the field of industrial maintenance.

## 2 Background

The selection of maintenance strategies plays a crucial role in the efficient management of industrial assets [2]. These strategies determine how maintenance activities are performed to ensure optimal operation and extend the useful life of assets [3].

The dataset was compiled to address the complexity of decision-making in industrial maintenance by providing data specifically designed for evaluating maintenance strategies under diverse conditions. It includes 12 criteria that are crucial for the selection of maintenance strategies, and the data was generated using Monte Carlo simulation techniques to ensure a broad range of scenarios. This simulation-based approach enables the capture of variability in maintenance decision factors, making the dataset highly comprehensive and applicable to a wide variety of industrial contexts. Compared to existing datasets, this collection offers a novel focus on scenario-based data generation, covering 1404 different scenarios. Many existing datasets in the field either lack this breadth or focus on single-case studies, limiting their generalizability. For example, the dataset presented by Pampana et al. [4] for building maintenance focuses on preventive and unplanned maintenance but does not generate diverse scenario-based data. Similarly, the dataset from Prieto Estacio et al. [5] on audio signals from DC motors is highly specialized, limiting its broader applicability to other industrial contexts.

Other datasets, such as the one by Fonseca et al. [6], focus on predictive maintenance using home appliances, but their data is heavily reliant on IoT and AI techniques, which makes it specific to smart devices rather than general industrial settings. Bravo et al. [7] provide a dataset on distribution transformers, but their focus is on grid operations, not maintenance strategy selection across a range of conditions. Lifsitch et al. [8] present a dataset based on fan coil motor vibrations, which, while valuable for motor health monitoring, lacks the flexibility to be applied across broader maintenance scenarios. Finally, Arain et al. [9] provide a dataset on railway track surface faults, but this dataset is designed for highly specific infrastructure applications and does not explore maintenance strategies beyond this niche.

By providing such a wide spectrum of simulated outcomes, our dataset fills a critical gap, offering data that can be used to explore a variety of maintenance strategies across different operational conditions.

Furthermore, while traditional datasets may emphasize heuristic or expert-driven approaches, this dataset is explicitly designed to be data-driven from the outset, without bias from prior maintenance methods or human expertise. This offers a neutral foundation that can be integrated with various decision-making tools, allowing for more objective and reproducible evaluations. The key contribution of this dataset lies in its ability to be easily replicated and adapted to other industrial maintenance contexts, providing a flexible tool for researchers and practitioners interested in optimizing maintenance strategies.

## 3 Data Description

**Fig. 1:**
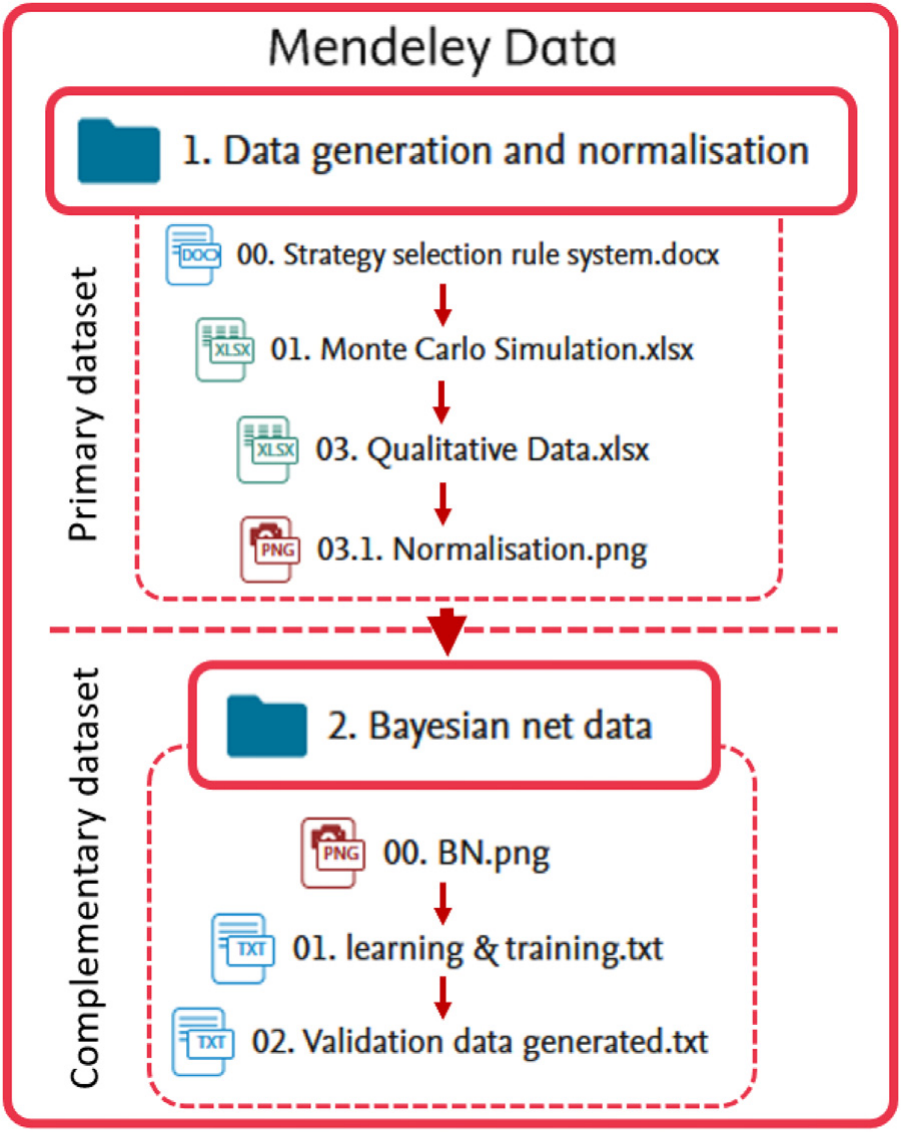
Graphical representation of the repository structure.

The dataset is organized into two folders. The first folder contains the primary dataset generated through Monte Carlo simulations, which includes the 1404 maintenance scenarios. The second folder provides supplementary materials, including the baseline results from the Bayesian network model. It is important to note that the folder labeled “Bayesian net data” is not part of the primary dataset. This folder can be retained as a baseline for comparison by researchers who wish to evaluate their methods against a traditional approach. However, these results are supplementary and not integral to the original data collection or generation process. Fig. 1 shows the graphical representation of the repository structure.

Folder 1: Data Generation and Normalization

**00. Strategy Selection Rules System.docx:** This document contains the rules table for maintenance strategy assignment and the criteria with their scales. Table 1 displays the selection rules for maintenance strategies.

**Table 1:** Strategy selection rule system.

Decision rules	Maintenance strategies
$C_{\mathrm{CORR}/\mathrm{PREV}}$ ˂ 3	Corrective (CM)
**C_PRED_≥2,5** OR C7≥3	Preventive (PM)
**C_PRED_≥2,5** AND (C4=1 OR C5=1)	Preventive by condition status (PSCM)
**C_PRED_≥3** AND C4=1	Predictive (PdM)
If C4 = 0 AND Cpred ˂ 2,4	Maintenance can be preventive or corrective, but not predictive
**C_PRED_≥3** AND (C5=1; C10=1; C11=1)	intelligent predictive (IPdM)

Where:

• **Predictive Maintenance Index**

(1) $$C_{PRED}=\frac{C1+C2+C3+C6+C8+C12}{6}$$

• **Corrective/Preventive Maintenance Index**

(2) $$C\frac{CORR}{PREV}=\frac{C_{Lost}+C_{Failure}}{6}\;$$

Where:

(3) $$C_{Lost}=C1+C2+C6$$

(4) $$C_{Failure}=C7+C8+C9$$

Evaluation criteria:

C1: Machine purchase costs

C2: Costs due to production losses

C3: Absence of duplicate and spare Machine parts

C4: Capacity to perform machine diagnostics using available instrumentation

C5: Access to sensors, Supervisory Control, and Data Acquisition (SCADA) systems

C6: Machine maintenance costs, including material and labour over a given period

C7: Lifetime loss due to disassembly

C8: Impact of failure on human safety and environment

C9: Economic impact of a machine breakdown

C10: Historical data availability

C11: Availability of artificial intelligence resources and skills

C12: Complexity of equipment

**01. Monte Carlo Simulation.xlsx:** This document simulates the rules system from the previous document using Excelʼs “IF” function to simulate rules and assign strategies. Criteria values were assigned using the “RAND.BETWEEN” function (x;y) according to each criterion's range, thereby assigning a maintenance strategy (Table 3).

**02. Monte Carlo-generated data.xlsx:** This dataset includes 1404 randomly simulated scenarios for learning and training, generated by Monte Carlo. This comprehensive set of scenarios enhances the performance of BN or other learning algorithms.

**03. Qualitative data.xlsx:** Normalization of Monte Carlo simulation data. This document replaces numeric values with qualitative ones to process data in the GeNIe software (see document “03.1. Normalization.png” or Table 2).

**Table 2:** Coefficients parameterisation.

Ccorr/prev	
Over 3	High
less than 3	Low
Cpredictive	
Over 3	High
Between 2.5 and 2.9	Medium
less than 2.4	Low
C-Lost and C-Failure	
Very High	13–15
High	10–12
Medium	7–9
Low	4–6
Very Low	1–3
5-point scale criteria	
Very low	1
Low	2
Medium	5
High	4
Very High	5

**Table 3:** Non-parameterised database fragment.

C1	C2	C3	C4	C5	C6	C7	C8	C9	C10	C11	C12	C-Predictive	C-Lost	C-Failure	Cprev/correc	CM	PM	PCSM	PdM	IPdM
2	2	2	1	1	5	5	2	5	1	1	2	2.5	9	12	3.5	No	Yes	Yes	No	No
5	5	4	0	0	1	3	4	1	0	0	4	3.8	11	8	3.2	No	Yes	No	No	No
3	3	3	0	0	4	4	1	2	0	0	1	2.5	10	7	2.8	Yes	Yes	No	No	No
4	4	4	1	0	1	5	2	3	1	0	5	3.3	9	10	3.2	No	Yes	Yes	Yes	No
2	4	2	1	0	4	1	5	4	0	0	5	3.7	10	10	3.3	No	Yes	Yes	Yes	No
5	4	3	0	1	2	2	5	3	0	1	3	3.7	11	10	3.5	No	Yes	Yes	No	No
2	5	3	1	0	5	1	4	4	0	1	1	3.3	12	9	3.5	No	Yes	Yes	Yes	No
5	4	4	0	1	4	3	2	5	1	1	4	3.8	13	10	3.8	No	Yes	Yes	No	Yes
5	5	5	0	1	4	3	1	1	0	1	2	3.7	14	5	3.2	No	Yes	Yes	No	No
5	1	4	0	0	1	4	2	3	1	0	2	2.5	7	9	2.7	Yes	Yes	No	No	No
2	2	5	1	1	2	3	1	5	0	1	3	2.5	6	9	2.5	Yes	Yes	Yes	No	No
2	4	4	1	1	2	2	3	1	1	1	2	2.8	8	6	2.3	Yes	Yes	Yes	No	No
5	1	2	1	0	2	2	4	1	0	1	4	3.0	8	7	2.5	Yes	Yes	Yes	Yes	No
3	1	4	1	1	5	5	1	3	1	1	3	2.8	9	9	3.0	No	Yes	Yes	No	No
4	1	5	0	1	4	5	4	5	1	1	5	3.8	9	14	3.8	No	Yes	Yes	No	Yes
1	4	1	0	1	3	5	1	3	0	1	1	1.8	8	9	2.8	Yes	Yes	No	No	No
1	5	2	0	1	2	2	3	3	1	1	4	2.8	8	8	2.7	Yes	Yes	Yes	No	No
4	5	2	1	1	5	3	1	4	0	1	2	3.2	14	8	3.7	No	Yes	Yes	Yes	No
5	4	1	0	1	4	2	5	4	0	1	4	3.8	13	11	4.0	No	Yes	Yes	No	No
2	5	3	1	0	4	4	5	5	0	0	2	3.5	11	14	4.2	No	Yes	Yes	Yes	No

**03.1. Normalization.png:** Visualization of scales

## 4 Experimental Design, Materials and Methods

### 4.1 Experimental objective

The primary objective of this experiment is to generate a comprehensive dataset for evaluating industrial maintenance strategies. The following sections outline the materials used, the experimental design, and the methods employed to create and collect the data.

### 4.2 Experimental design

**Fig. 2:**
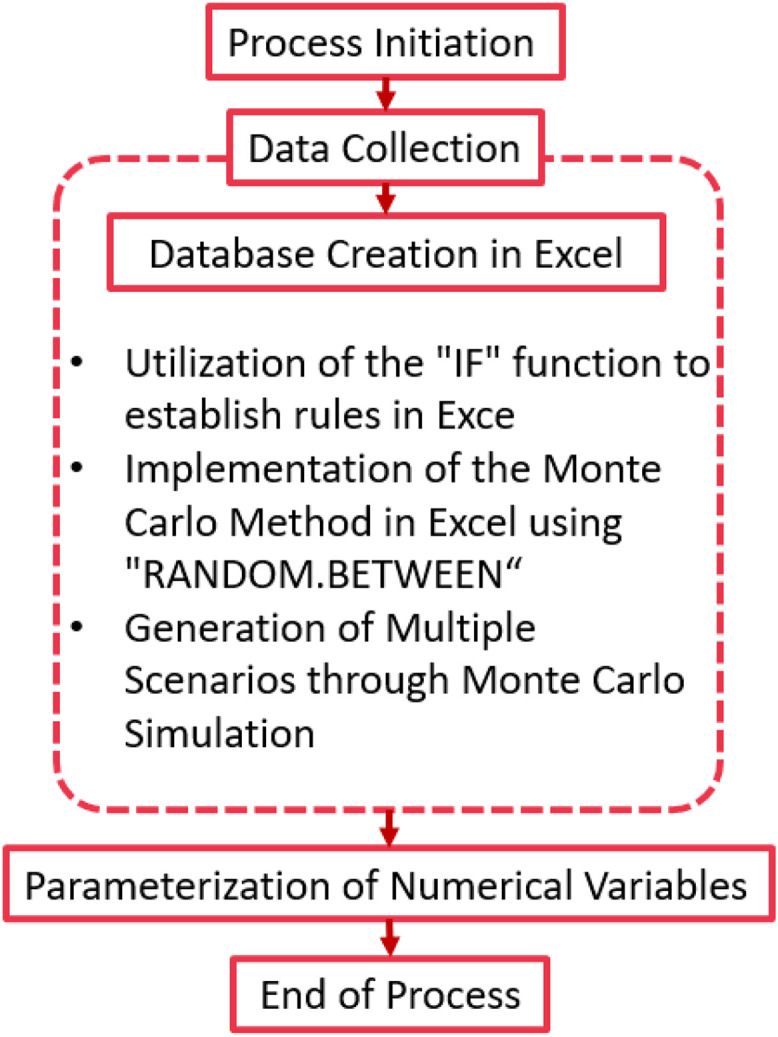
Flow chart for data collection and processing.

The experimental design focuses on the systematic collection and generation of data through Monte Carlo simulations. The aim is to create a dataset that reflects a wide range of maintenance scenarios by varying key parameters related to maintenance strategies. Fig. 2 presents the flow chart that outlines the data generation and processing steps.

### 4.3 Materials

**Software:**

Microsoft Excel: For data organization and simulation.

**Data:**

“00. Strategy selection rules system.docx”

“01. Monte Carlo Simulation.xlsx”

“02. Monte Carlo-generated data.xlsx”

“03. Qualitative data.xlsx”

**Excel Functions:**

"IF" Function: To establish rules.

"RANDOM.BETWEEN" Function: For Monte Carlo simulation.

## 5 Methods

### 5.1 Monte Carlo simulation

**Rule system**

**Explanation of Decision Rules:**

**Corrective Maintenance (CM):**

Rule: C_(CORR⁄PREV) < 3

Explanation: When the Corrective-to-Preventive Maintenance Index (C_CORR/PREV) is less than 3, corrective maintenance is selected. This indicates a scenario where the combined impact of losses and failures is relatively low, suggesting that corrective actions are more feasible or cost-effective.

**Preventive Maintenance (PM):**

Rule: C_PRED ≥ 2.5 OR C7 ≥ 3

Explanation: Preventive maintenance is chosen if the Predictive Maintenance Index (C_PRED) is greater than or equal to 2.5 or if the lifetime loss due to disassembly (C7) is significant (≥ 3). This ensures preventive measures are taken in situations with high predictive scores or substantial disassembly losses, mitigating potential issues proactively.

**Preventive by Condition Status Maintenance (PSCM):**

Rule: C_PRED ≥ 2.5 AND (C4=1 OR C5=1)

Explanation: This strategy is selected when the Predictive Maintenance Index (C_PRED) is at least 2.5 and either the capacity to perform machine diagnostics using available instrumentation (C4) or access to sensors and SCADA systems (C5) is present. This focuses on condition-based maintenance when diagnostic tools or data acquisition systems are available, ensuring maintenance actions are based on real-time data and condition monitoring.

**Predictive Maintenance (PdM):**

Rule: C_PRED ≥ 3 AND C4=1

Explanation: Predictive maintenance is implemented when the Predictive Maintenance Index (C_PRED) is at least 3 and the capacity to perform machine diagnostics (C4) is available. This indicates a high predictive score combined with effective diagnostics, justifying the use of advanced predictive techniques to anticipate and prevent potential failures.

**Conditional Preventive or Corrective Maintenance:**

Rule: If C4 = 0 AND C_PRED < 2.4

Explanation: In scenarios where diagnostics capacity (C4) is absent and the Predictive Maintenance Index (C_PRED) is less than 2.4, the maintenance strategy can be either preventive or corrective, but not predictive. This flexibility allows for a tailored approach depending on the specific context and condition, ensuring that maintenance actions are suitable given the lack of diagnostic capabilities and low predictive scores.

**Intelligent Predictive Maintenance (IPdM):**

Rule: C_PRED ≥ 3 AND (C5=1; C10=1; C11=1)

Explanation: Intelligent predictive maintenance is selected when the Predictive Maintenance Index (C_PRED) is at least 3 and there is access to sensors and SCADA systems (C5), historical data (C10), and artificial intelligence resources and skills (C11). This sophisticated rule combines high predictive scores with comprehensive data and AI resources, necessitating the use of intelligent and advanced predictive maintenance strategies. This ensures that maintenance decisions are data-driven, leveraging historical and real-time data alongside AI capabilities to optimize predictive maintenance actions.

**Step 1: Database Creation in Excel and Assignment of Values to Criteria Using the Monte Carlo Method**

The Monte Carlo simulation is essential for generating diverse scenarios due to the complexity and number of criteria and indices involved in the data article. With 12 evaluation criteria and 2 indices, each ranging in value from one to five, the possible combinations are extensive. Manually creating these scenarios would be impractical and time-consuming. The Monte Carlo method allows for efficient generation of a vast array of scenarios by randomly assigning values to each criterion, thereby covering a wide spectrum of possible situations.

This approach is particularly crucial for models training, by simulating 1404 different scenarios, the data article ensures a comprehensive coverage of potential maintenance situations, reflecting the variability and complexity found in actual industrial operations. This extensive scenario analysis provides a robust framework for evaluating maintenance strategies, making the simulated data highly representative of real-world conditions. Consequently, the use of Monte Carlo simulation not only streamlines the generation of comprehensive scenarios but also enhances the model's ability to generalize and infer from a broad range of potential real-world conditions.

A database was developed in Excel where each column represents a node and each row represents different states of the node, organizing the necessary information for maintenance strategies selection. The nodes represented were the 12 criteria, the coefficients, and the maintenance strategies to be selected.

Use of the “IF” Function: The “IF” function was used to translate the rules from Table 1 into the spreadsheet format, automating the assignment of maintenance strategies based on the criteria values. The coefficients C-Corrective/Preventive and C-Predictive were also calculated according to their respective formulas, as well as C-lost and C-failure.

Implementation of the Monte Carlo Method: The “RANDOM.BETWEEN” function was used to assign random values to the criteria, simulating a wide range of possible combinations to evaluate maintenance strategies.

Generation of Multiple Scenarios: The simulation of various combinations allowed for the coverage of a broad spectrum of scenarios, providing a comprehensive view of the potential strategies to consider. With the completion of this step, the raw data that constitutes both “01. Monte Carlo Simulation.xlsx” and “02. Monte Carlo-generated data.xlsx” was obtained. In total, 1404 scenarios were simulated. The first document, “01. Monte Carlo Simulation.xlsx”, contains the functions “IF” and “RANDOM.BETWEEN” used to generate the raw data. The second document, “02. Monte Carlo-generated data.xlsx”, presents the selection of these generated scenarios.

**Step 2: Parameterization**

Parameterization transformed numerical variables into qualitative or categorical ones. The coefficients C-predictive, C-loss, C-failure, and C-corrective/Preventive were converted to their respective scales, as were the 12 evaluated criteria. The raw data that constitutes the document “02. Monte Carlo-generated data.xlsx” was used for this parameterization. The parameterized data constitutes the document "03. Qualitative data.xlsx which contains the parameterized version of the dataset, which organizes the data into qualitative scales for ease of use in further analysis.

**Step 3: Data Documentation**

The complete set of simulated data is organized into three main documents:

• “01. Monte Carlo Simulation.xlsx,” which contains the raw data generation functions.
• “02. Monte Carlo-generated data.xlsx,” which presents the finalized data for the 1404 scenarios.
• “03. Qualitative data.xlsx” which contains the parameterized version of the dataset.

These documents provide the foundation for replicating the data generation process, offering researchers a flexible dataset that can be adapted to different industrial contexts.

## Limitations

The nature of generating random scenarios using the Monte Carlo simulation method may be a limitation of the data described in this article. Although this technique has the advantage of creating a wide range of potential combinations for evaluating maintenance strategies, the randomness of the values introduces variability in the dataset. Although 1404 randomized scenarios were used in the evaluation, this variability is inherent in the random assignment of values.

## Ethics Statement

This work did not involve any human or animal subjects, nor data from social media platforms.

## Credit Author Statement

**Raúl Torres-Sainz:** methodology, software, writing - original draft preparation, and data curation; **Leandro L. Lorente-Leyva:** formal analysis, writing - review and editing, resources, and project administration; **Yorley Arbella-Feliciano:** investigation, visualization, and funding acquisition; **Carlos Alberto Trinchet-Varela:** conceptualization, methodology, formal analysis and writing - review and editing; **Lidia María Perez-Vallejo:** data curation, project administration, writing - original draft preparation, and writing - review and editing; **Roberto Pérez-Rodríguez:** investigation, methodology, supervision, and writing - review and editing. All authors have read and agreed to the published version of the manuscript.

## Data Availability

Mendeley DataBayesian network analysis for maintenance strategy selection (Original data).
